# Pulmonary nodules associated with JAK inhibitor therapy in rheumatoid arthritis: A case report

**DOI:** 10.7196/AJTCCM.2021.v27i3.116

**Published:** 2021-10-04

**Authors:** N Pannell, D Joseph, M M T M Ally, N M Bida, G R Tintinger

**Affiliations:** Department of Internal Medicine, Faculty of Health Sciences, University of Pretoria, South Africa

**Keywords:** Janus kinase inhibitors, opportunistic infections

## Abstract

Patients with rheumatoid arthritis (RA) may receive Janus kinase (JAK) inhibitors to achieve optimal control of their disease. We report a case of a patient who received a selective JAK1 inhibitor and subsequently developed multiple pulmonary nodules with cavitation. Biopsies confirmed the presence of cryptococcosis and the patient responded well to anti-fungal therapy.

## Background

Treatment of rheumatoid arthritis (RA) has significantly improved over the last decade. Many patients require multiple drugs to maintain adequate control of their disease and a large number of treatment options are currently available.

Janus kinase (JAK) inhibitors block the activity of one or more of the JAK family of enzymes (JAK1, JAK2, JAK3 and tyrosine kinase 2) and interfere with the JAK-signal transducer and activator of transcription (STAT) pathway.^[1]^ More than 50 cytokines signal via the JAK/STAT pathway to induce inflammation, direct haematopoiesis and control the immune system.

## Case

Mrs TN is a 61-year-old woman from Gauteng Province in South Africa (SA). Informed consent was obtained from the patient. She is a patient with RA and at the time of presentation had been managed with disease-modifying anti-rheumatic drugs (DMARDs) including methotrexate and oral corticosteroids. In addition, she was enrolled in a biological therapeutic trial using a selective JAK1 inhibitor. She was not known to be HIV-infected and her known comorbid conditions included hypertension and dyslipidaemia.

She was initially admitted to hospital in May 2019 with a diagnosis of multi-lobar pneumonia, for which she received empiric antibiotic therapy and was later discharged. Subsequent follow-up at the rheumatology clinic revealed worsening lung infiltrates, despite a lack of symptoms and clinical complaints. She was referred to the pulmonology clinic for further assessment, and her clinical examination was noted to be remarkably normal with an oxygen saturation of 93% on room air. Pulmonary function testing showed a post bronchodilator forced expiratory volume in 1 second to forced vital capacity ratio (FEV_1_ /FVC) of 0.79, total lung capacity of 4.78 L (109.26% of predicted) and a diffusion capacity for carbon monoxide per alveolar volume (DLCO/VA) of 0.34 nmol/ min/kPa/L (21% of predicted). The low DLCO may be due to a ventilation/perfusion (V/Q) mismatch secondary to underlying lung disease.

**Fig. 1:**
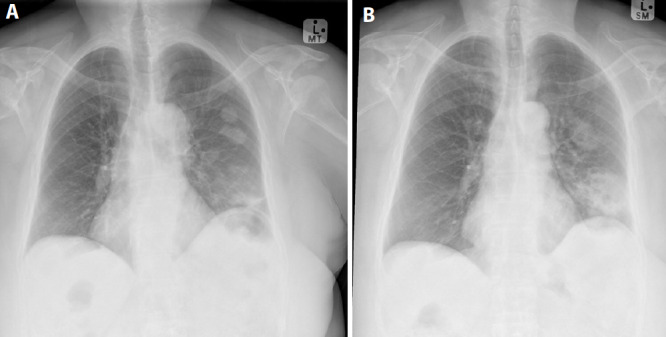
Initial (A) and repeat (B) chest radiograph.

Her initial chest radiograph from May 2019 showed 2 discrete solid lung nodules in the left mid to upper lung zones measuring 15 mm × 17 mm and 17 mm × 19 mm, respectively. In addition, the left lower zone revealed an ill-defined area of ground glass opacification (Fig. 1A). A repeat chest radiograph in July 2019 revealed that the lesions had increased in size (Fig. 1B).

**Fig. 2:**
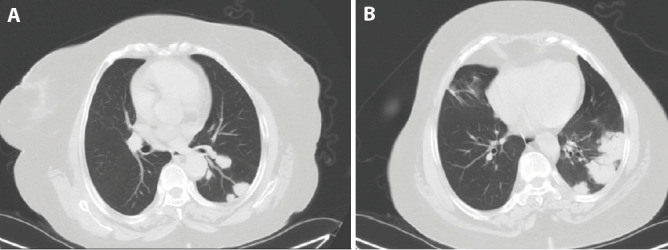
Computed tomography scan of the chest.

A computed tomography (CT) scan of the chest revealed multiple, well defined, peripheral and patchy air space opacifications with airbronchograms involving the left lower lobe and abutting the oblique fissure. This was associated with pulmonary nodules and left pleural thickening (Figs 2A and B). Initial blood results revealed a C-reactive protein (CRP) of 5 mg/L, erythrocyte sedimentation rate (ESR) of 20 mm/hr and a normal full blood count. Sputum investigations were negative for tuberculosis.

**Fig. 3:**
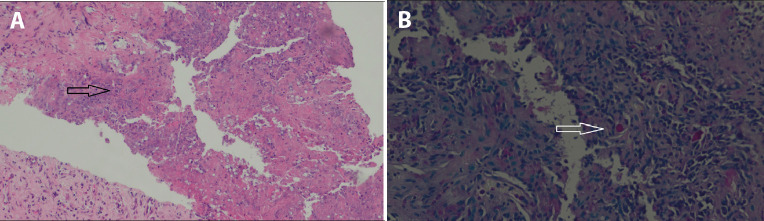
A photomicrograph of an H & E stain lung showing necrotising granulomatous inflammation and fibrous thickening of the pleura (A), 20× magnification. A photomicrograph of a section stained with Alcian Blue and PAS (ABPAS) showing fungal elements with morphological features of *Cryptococcus* (B).

Metastases should be bilateral, and the evolution and doubling time is unlike malignancy. In addition, infections, pulmonary infarctions and vasculitic lesions could be considered, but the patient’s normal CRP and ESR were not consistent with an acute inflammatory state. Owing to the peripheral nature of these lesions, she underwent a CT-guided transthoracic lung biopsy, which revealed fibrous connective tissue with evidence of chronic necrotising granulomatous inflammation and fungal organisms with the morphological features of *Cryptococcus* (Figs 3A and B).

Ziehl-Neelsen staining was negative for acid- and alcohol-fast bacilli. A diagnosis of pulmonary cryptococcosis was made.

### Treatment and outcome

**Fig. 4:**
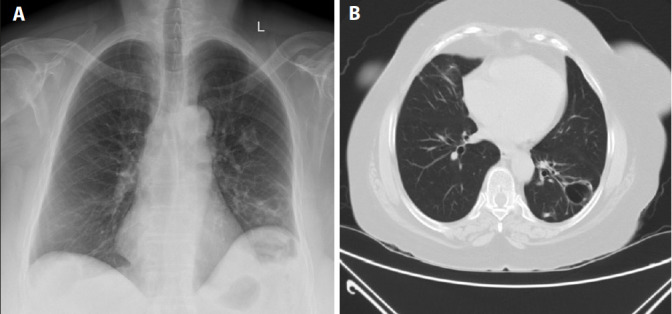
Repeat chest radiograph (A) and computed tomography images 6 months after initiation of therapy (B).

The JAK1 inhibitor was discontinued and the patient was started on oral fluconazole at a dose of 400 mg 12-hourly for the duration of 1 month. Follow-up chest radiography revealed partial resolution of the lesions noted previously (Fig. 4A). Fluconazole 200 mg twice daily for 3 months and then 200 mg daily thereafter was continued. Subsequent repeat CT imaging done 6 months after initiation of therapy revealed resolution of lung nodules and masses, with some residual fibro-cavitary lung changes remaining in areas previously involved by mass formation (Fig. 4B). The patient has fortunately maintained a stable clinical condition and remains well, without deterioration of her lung functions and her RA has remained stable.

## Discussion

Selective JAK1 inhibitors are indicated for the treatment of moderately to severely active RA in adults who have had an inadequate response or intolerance to conventional synthetic DMARDs and biologic DMARDs.

Although the use of oral corticosteroids may predispose patients to opportunistic infections, the most common adverse events associated with JAK1 inhibitors are nasopharyngitis and serious infections including opportunistic infections and herpes zoster. A spectrum of serious opportunistic infections that may occur and lead to hospitalisation or death include bacteria (e.g. *Mycobacterium tuberculosis*), viruses and fungi such as *Pneumocystis jirovecii*. ^[2]^ In addition, invasive fungal infections due to *Cryptococcus* spp. have also been reported.^[3]^

Protein kinase inhibitors block signalling pathways involving multiple cytokines and growth factors with the potential to cause significant immune disturbances. Downregulation of cytokine signalling with JAK inhibitors decreases the activation and infiltration of natural killer cells and macrophages, interfering with phagocytic activity. Opportunistic fungal infections can cause considerable morbidity in immunocompromised patients. Cryptococcosis is an opportunistic disease usually presenting as meningitis or meningoencephalitis.

Pulmonary cryptococcosis is under-diagnosed and ranges from asymptomatic infection to pneumonia and respiratory failure.^[4]^ *Cryptococcus* is found in soil contaminated by pigeon droppings and has also been found from the heartwood of several trees. Exposure is common and most people have been exposed by the age of 5 years.^[4]^

Clinical manifestations vary among patients, with immunocompetent patients being asymptomatic.^[5]^ The degree of immunosuppression influences the natural history of pulmonary cryptococcosis.^[6]^ Typical symptoms are cough, sputum production, chest pain, fever, dyspnoea, night sweats and haemoptysis.^[5]^

Radiological findings are influenced by the degree of immunosuppression.^[4]^ The most common findings in immunocompetent patients are pulmonary nodules or masses.^[5]^ Immunocompromised patients have a wider variety of radiological abnormalities with a higher incidence of diffuse interstitial infiltrates, segmental or lobar consolidation and cavitation and a lower proportion of pulmonary nodules.^[4–6]^ The clinical findings and radiographical features observed in this patient are in keeping with those usually observed in persons with less-pronounced immunosuppression. Tissue biopsy and/ or a positive fungal culture are the most accurate means of diagnosis.^[5]^ Serum cryptococcal antigen testing is more likely to be positive in disseminated disease in an immunocompromised patient.^[6]^

The goals for treatment of pulmonary cryptococcosis are to treat symptomatic infection and prevent dissemination. Patients with mild to moderate symptoms or asymptomatic patients with positive cultures should be treated with fluconazole, 400 - 800 mg daily for up to 10 weeks and then 200 mg daily for 6 months or more. Patients with severe disease should receive induction therapy with amphotericin B (0.7 mg/kg/d) with or without flucytosine and maintenance treatment with fluconazole.^[4]^ The decision to discontinue maintenance treatment should consider host immunity and clinical and radiological findings on a case-by-case basis.^[6]^

## Conclusion

Patients receiving a selective JAK1 inhibitor may develop pulmonary cryptococcal infections. A high index of suspicion, appropriate and timely diagnostic and therapeutic interventions may be necessary to manage these patients successfully.
